# Letter to editor

**DOI:** 10.1186/s44158-021-00024-5

**Published:** 2021-12-09

**Authors:** Antonio Messina, Maurizio Cecconi

**Affiliations:** 1 IRCCS Humanitas Research Hospital, Rozzano, Milano, Italy; 2 Department of Biomedical Sciences, Humanitas University, Pieve Emanuele, Milano, Italy

Dear Editor,

In a recent published paper, our group investigated the pharmacodynamic effect of a fluid challenge (FC) of a 4 ml/kg of crystalloids infused over 10 (FC_10_) or over 20 min (FC_10_), with the purpose of assess whether the fluid responsiveness [defined as an increase in the stroke volume index (SVI) ≥ 10%] was affected by the time of infusion [1].

Considering the effect of the different time of infusion on both pressure and flow variables, in a secondary analysis of the paper, we calculated the percent changes of systolic arterial pressure (ΔSAP) and SVI (ΔSVI) from baseline to the half of the infusion of FC_10_ and FC_20_ and assessed the correlation by linear regression and the reliability of SAP changes in predicting fluid responsiveness by considering the area under (AUC_ROC_) the receiver operating characteristic (ROC) [95% confidence interval (95% CI)]. The grey zones for all the statistically significant ROC curves were also calculated considering the low cut-off value including 90% of negative FC responses and a high cut-off value predicting positive FC in 90% of cases [2].

**Fig. 1:**
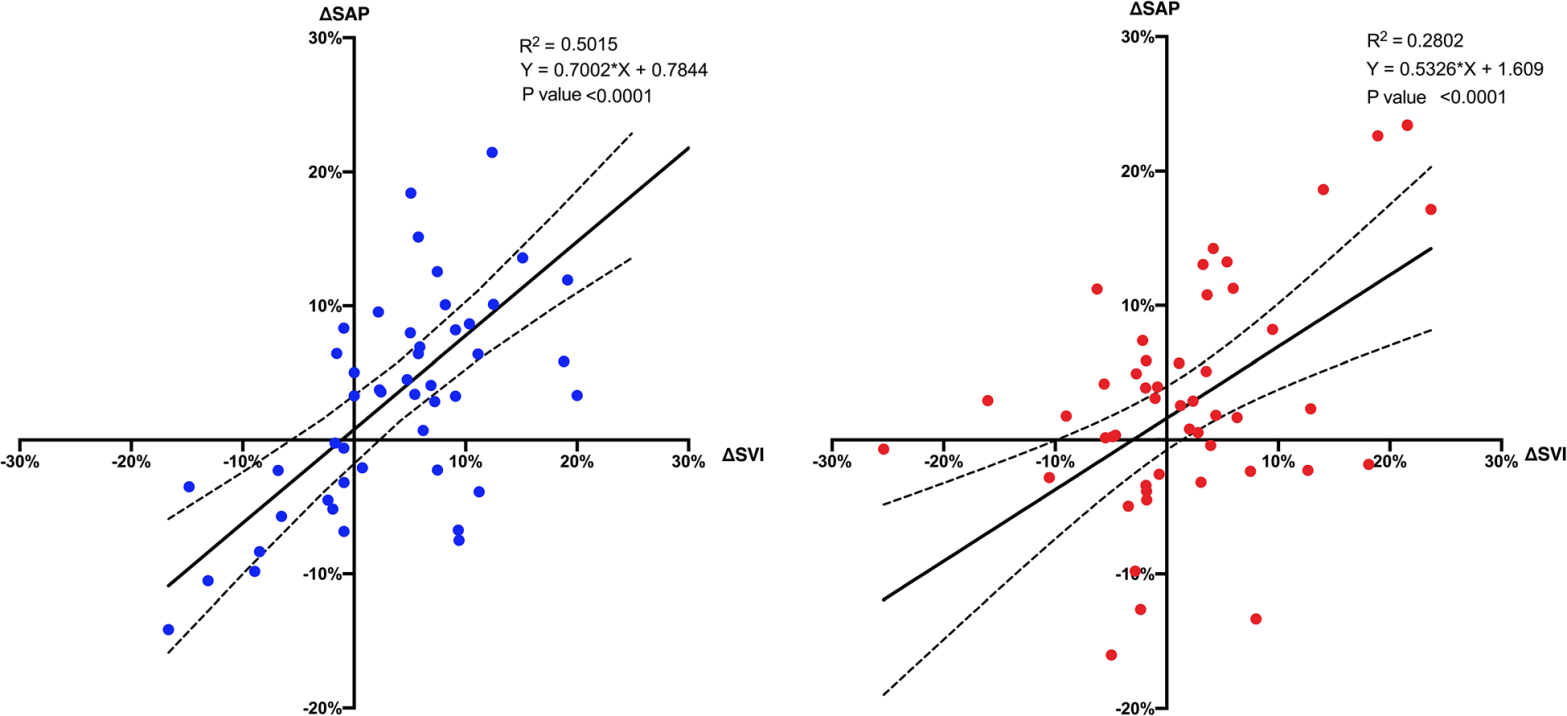
Linear regression between the changes in SVI (ΔSVI) and in SAP (ΔSAP) after ½ fluid challenge administration in the FC_10_ (blue dots, left panel) and FC_20_ (red dots, right panel). Dashed lines represent 95% confidence intervals for the regression line (solid line)

There was a significant positive correlation between ΔSAP and ΔSVI from baseline to ½ FC administration, during both FC_10_ (*r*^2^ = 0.50; *p* < 0.0001; slope = 0.70 ± 0.10) and FC_20_ (*r*^2^ = 0.28; *p* = 0.01; slope = − 0.53 ± 0.12) administrations (Fig. 1). However, the ROC curve of the changes in SAP after 1/2 FC_10_ was significant (*p* = 0.01) [AUC_ROC_ = 0.72 (95% CI 0.55–0.85); gray zone 10%/0%]. On the contrary, the ROC curve of the changes in SAP after ½ FC_20_ was not statistically significant (*p* = 0.11). Our results show a positive moderate linear correlation (*r*^2^ = 0.50) between for ΔSAP and ΔSVI FC_10_. The associated ROC curve constructed showed that ΔSAP > 10% is highly suggestive of FC response (i.e., sensitivity > 90%), whereas ΔSAP = 0 is highly suggestive of no response (i.e., specificity > 90%). On the contrary, the linear correlation of FC_20_ was weak (*r*^2^ = 0.28), and the ROC curve was insignificant.

The interplay between SAP and SVI is based on a complex balance between cardiac factors, arterial load, and resistance [3, 4]. In fact, the physiological relationship between pressure and flow variables is not linear, and SAP changes should not be used as a perfect surrogate for SVI to predict the effect of FC [5, 6]. Nevertheless, ΔSAP, only after FC_10_, still maintains clinical utility, suggesting that no increase after ½ FC is associated with no fluid responsiveness, whereas an increase of at least 10% is associated to fluid responsiveness. In contexts of low resources or when a SVI monitoring is not available, our results may provide practical cut-offs to guide fluid optimization in elective surgical patients. This finding, however, could be partially dependent on the intrinsic mathematical coupling between pressure and flow variables changes after the fluid infusion, since the MostCare® system is based on the high sample rate analysis of the arterial waveform.

Respectfully Yours.
